# Characterization of the Commercially-Available Fluorescent Chloroquine-BODIPY Conjugate, LynxTag-CQ_GREEN_, as a Marker for Chloroquine Resistance and Uptake in a 96-Well Plate Assay

**DOI:** 10.1371/journal.pone.0110800

**Published:** 2014-10-24

**Authors:** Cheryl C. Y. Loh, Rossarin Suwanarusk, Yan Quan Lee, Kitti W. K. Chan, Kit-Ying Choy, Laurent Rénia, Bruce Russell, Martin J. Lear, François H. Nosten, Kevin S. W. Tan, Larry M. C. Chow

**Affiliations:** 1 Department of Microbiology, Yong Loo Lin School of Medicine, National University of Singapore, Singapore, Singapore; 2 Singapore Immunology Network, Agency for Science Technology and Research, Biopolis, Singapore, Singapore; 3 NUS Graduate School for Integrative Sciences and Engineering, National University of Singapore, Singapore, Singapore; 4 Department of Applied Biology and Chemical Technology, The State Key Laboratory of Chirosciences, The Hong Kong Polytechnic University, Hung Hom, Kowloon, Hong Kong SAR, PR China; 5 Department of Chemistry, Graduate School of Science, Tohoku University, Aza Aramaki, Aoba-ku, Sendai, Japan; 6 Centre for Tropical Medicine, Nuffield Department of Medicine, University of Oxford, Oxford, United Kingdom; 7 Shoklo Malaria Research Unit, Mahidol-Oxford Tropical Medicine Research Unit, Faculty of Tropical Medicine, Mahidol University, Mae Sot, Thailand; 8 Centre for Tropical Medicine, University of Oxford, Churchill Hospital, Oxford, United Kingdom; Université Pierre et Marie Curie, France

## Abstract

Chloroquine was a cheap, extremely effective drug against *Plasmodium falciparum* until resistance arose. One approach to reversing resistance is the inhibition of chloroquine efflux from its site of action, the parasite digestive vacuole. Chloroquine accumulation studies have traditionally relied on radiolabelled chloroquine, which poses several challenges. There is a need for development of a safe and biologically relevant substitute. We report here a commercially-available green fluorescent chloroquine-BODIPY conjugate, LynxTag-CQ_GREEN,_ as a proxy for chloroquine accumulation. This compound localized to the digestive vacuole of the parasite as observed under confocal microscopy, and inhibited growth of chloroquine-sensitive strain 3D7 more extensively than in the resistant strains 7G8 and K1. Microplate reader measurements indicated suppression of LynxTag-CQ_GREEN_ efflux after pretreatment of parasites with known reversal agents. Microsomes carrying either sensitive- or resistant-type PfCRT were assayed for uptake; resistant-type PfCRT exhibited increased accumulation of LynxTag-CQ_GREEN_, which was suppressed by pretreatment with known chemosensitizers. Eight laboratory strains and twelve clinical isolates were sequenced for PfCRT and Pgh1 haplotypes previously reported to contribute to drug resistance, and *pfmdr1* copy number and chloroquine IC_50_s were determined. These data were compared with LynxTag-CQ_GREEN_ uptake/fluorescence by multiple linear regression to identify genetic correlates of uptake. Uptake of the compound correlated with the logIC_50_ of chloroquine and, more weakly, a mutation in Pgh1, F1226Y.

## Introduction

Despite years of intense global effort to eradicate it, malaria is still one of the deadliest infectious diseases, killing more than 600 000 people in 2010 alone [1], [2]. The severest form of malaria is caused by the protozoan parasite *Plasmodium falciparum*. Chloroquine (CQ), once a spectacularly successful antimalarial drug, was first discovered by the German chemist Johann Andersag but was mistakenly thought to be too toxic for therapeutic purposes, an incident which became known as “the resochin error” (resochin being the name given to the compound by Andersag) [3], [4]. CQ was so effective that it inspired optimism for the eradication of malaria. However, resistance soon arose, first appearing along the Thai-Cambodian border in the 1950s. By the 1970s, CQ resistance had spread throughout the world [5], [6]. This resistance is generally attributed to mutations in the *pfcrt* (*P. falciparum* chloroquine resistance transporter) gene, which codes for a transporter situated on the membrane of the parasite digestive vacuole (DV).

During parasite development in the intraerythrocytic cycle, haemoglobin is digested in the DV and the toxic heme moiety is released, which the parasite crystalizes into non-toxic hemozoin [7]. CQ is generally thought to kill the parasite by inhibiting the formation of hemozoin and thus preventing the detoxification of free heme [8]–[10]. In wild-type parasites CQ diffuses through the DV membrane and is diprotonated in the acidic environment of the DV, acquiring a net positive charge which prevents it from escaping the DV; however, mutant PfCRT found in CQ-resistant parasites effluxes this charged CQ out of the DV, removing it from its site of action [11]. Although the current first line artemisinin-combination therapies are effective in clearing parasitaemia, resistance against artemisinins has emerged [12]–[17]. There is therefore an urgent need to develop novel antimalarial strategies. Several research groups, including our own, have tried different approaches to tackle the problem of CQ resistance by either reversing CQ resistance with a PfCRT inhibitor or synthesizing “reversed” CQ analogues that cannot be effluxed by PfCRT [18]–[24]. The ultimate goal is to reintroduce CQ as a viable treatment for malaria. Both development of PfCRT inhibitors and synthesis of “reversed” CQ analogues require a sensitive assay for CQ uptake which is typically performed by the use of radiolabelled CQ [22], [25]–[28]. Such methods are difficult to adopt in a high-throughput screen and may raise concerns of safety. To overcome this technical difficulty, fluorescent derivatives of chloroquine have recently been developed and used for this purpose; fluorophores used include 6-(N-(7-nitrobenz-2-oxa-1, 3-diazol-4-yl)amino)hexanoic acid (NBD) [29], coumarin [21], [30], and 4, 4-difluoro-4-bora-3a, 4a-diaza-*s*-indacene (BODIPY) [31].

BODIPY derivatives typically exhibit strong fluorescence and are relatively inert in biological conditions [32]. Furthermore, their maximum emission wavelengths are in the green-red region [32], allowing them to be used with many DNA dyes that fluoresce blue, such as the DAPI and Hoechst stains. These properties make BODIPY a promising candidate as a marker for CQ uptake in *P. falciparum*. We therefore present here the characterization of a commercially-available BODIPY-CQ conjugate, LynxTag-CQ_GREEN_, in several laboratory strains and clinical isolates.

## Methods

### Parasite culture and synchronization

*P. falciparum* laboratory strains 3D7 (MRA-102), K1 (MRA-159), 7G8 (MRA-154), HB3 (MRA-155), CS2 (MRA-96), T9-94 (MRA-153), and Dd2 (MRA-156) were obtained from MR4, ATCC Manassas Virginia. Strain T9/96 was obtained from The European Malaria Reagent Repository. A further twelve clinical isolates were collected from the Mae Sot district, Tak Province, in northwest Thailand at the Shoklo Malaria Research Unit; these isolates are prefixed ‘SMRU’. Parasites were continuously cultured in complete malaria culture media (MCM) consisting of RPMI 1640 (Life Technologies) supplemented with 0.5% (w/v) Albumax I (Invitrogen), 0.005% (w/v) hypoxanthine, 0.03% (w/v) L-glutamate, 0.25% (w/v) gentamycin, with human erythrocytes at 2.5% haematocrit. Cultures were gassed with a mixture of 3% CO_2_, 4% O_2_ and 93% N_2_ and incubated at 37°C. Synchronization of parasite cultures was performed by resuspending erythrocytes in 5% (w/v) D-sorbitol and incubating at 37°C for 10 min, after which the erythrocytes were washed twice, resuspended in MCM and returned to culture conditions. Thin Giemsa smears were made before each experiment to determine parasitemia and parasite stage.

### Compound preparation

For work involving parasites, chlorpheniramine maleate salt, chlorpromazine hydrochloride, desipramine hydrochloride, promethazine hydrochloride, verapamil hydrochloride and CQ diphosphate (all from Sigma-Aldrich) were dissolved in PBS to a working concentration of 1 mM. LynxTag-CQ_GREEN_ (BioLynx Technologies, Singapore; hereafter abbreviated to ‘CQ_GREEN_’) was dissolved in DMSO to the same concentration. All compounds were stored at −20°C and protected from light. For microsome uptake assays, methiothepin mesylate salt, metergoline, loperamide hydrochloride, octoclothepin maleate salt, mibefradil dihydrochloride hydrate, L703,606 oxalate salt hydrate, and chlorprothixene hydrochloride (all from Sigma-Aldrich) were dissolved in DMSO to 10 mM and stored at 4°C. Verapamil hydrochloride, adenosine triphosphate (ATP), and CQ diphosphate (all from Sigma-Aldrich) were dissolved in water to 7.5 mM, 50 mM and 0.1 M respectively and stored at −20°C. Tritiated CQ (^3^H-CQ; from Moravek Biochemicals and Radiochemicals) was diluted in water to 5.32 µM and stored at −20°C; specific activity was 4.7 Ci/mmol.

### Reinvasion half-maximal inhibitory concentration (IC_50_)

Synchronized ring-stage cultures at 1–2% parasitemia, 1.25% haematocrit were incubated with either CQ or CQ_GREEN_ at a range of concentrations for 48 h in 96-well flat-bottomed plates at culture conditions. Following this, cells were stained with 1 µg/ml of Hoechst 33342 (Invitrogen) for 30 min at 37°C, washed twice and resuspended in PBS. Parasitemia was then assessed with the CyAn ADP flow cytometer (Beckman Coulter). IC_50_s were determined by plotting the measurements in Graphpad Prism 5 using a variable slope logistic curve.

### Confocal imaging

200 µl cultures of 3D7 at 3% parasitemia, 1.25% haematocrit were incubated with CQ_GREEN_ for 2 h at 2 µM in a 96-well plate format. Erythrocytes were then washed twice and stained with Hoechst 33342 as previously. Wet mounts of stained parasites were visualized under ×100 magnification with the Fluoview FV1000 confocal microscope (Olympus). Hoechst and CQ_GREEN_ were excited at 405 nm and 488 nm with emissions captured at 430–470 nm and 505–525 nm respectively.

### Parasite CQ_GREEN_ uptake assay

Synchronized trophozoite-stage cultures at 3–5% parasitemia were resuspended in 200 µl of MCM with 2 µM of CQ_GREEN_ to 2.5% haematocrit in a 96-well plate format. The parasites were then incubated for 2 h at culture conditions, after which they were washed twice and resuspended in PBS. Cells were allowed to settle in a Nunc F96 MicroWell black non-treated polystyrene plate (Thermo Scientific) for 1 h. Fluorescence was then measured with the Infinite M200 microplate reader (Tecan) with excitation and emission wavelengths of 488 nm and 520 nm respectively. K1 chemoreversal assays were performed by pretreatment with 10 µM of the reversal agents for 30 min prior to the addition of CQ_GREEN_.

### Preparation of microsomes carrying PfCRT

PfCRT originating from *P. falciparum* strains Dd2 or 3D7 were expressed in *Pichia pastoris* KM71 and microsomes harvested as described previously [33]. Microsomal levels of PfCRT were determined by western blot with standard curves generated from blots of purified PfCRT.

### Uptake kinetics in microsomes

In order to assess the Michaelis-Menten kinetics of CQ_GREEN_ uptake by the microsomes, total or non-specific uptake was measured. Non-specific uptake was determined by pretreating microsomes with unlabelled CQ at 1000 times of the concentration of CQ_GREEN_ used, for 15 min at 37°C, before adding CQ_GREEN_; total uptake was determined without the pretreatment. Reactions were carried out in accumulation buffer (0.25 M sucrose, 10 mM Tris–HCl, 5 mM MgCl_2_, and 3 mM ATP, pH 7.5). Following this, microsomes were washed twice in accumulation buffer, then lysed in lysis buffer (0.75 M HCl, 1% Triton X-100, 77.5% isopropanol) on ice for at least one hour. Unlabelled CQ was excluded from the washing step as the addition of CQ was observed to cause displacement of CQ_GREEN_ from the microsomes, possibly due to the higher affinity of CQ for PfCRT. Measurement of the fluorescence intensity was performed using the FLUOstar Galaxy microplate reader (BMG Labtech) with excitation and emission wavelengths of 480 nm and 520 nm respectively. For each experiment, measurements were made in triplicate and the mean calculated. Specific uptake was then calculated by subtracting non-specific uptake from total uptake. Non-linear regression analysis with the Michaelis-Menten model (GraphPad Prism 5) was then applied to obtain the V_max_ and K_m_ of specific uptake.

### CQ_GREEN_ uptake in PfCRT microsomes

Unless stated otherwise, microsomes were incubated with 15 µM CQ_GREEN_ at 37°C for 15 min. For uptake inhibition assays, microsomes were pre-incubated with chemoreversal compounds [21] for 15 min at 37°C prior to the addition of CQ_GREEN_. All data presented are specific uptake based on the calculations stated above.

### ^3^H-CQ uptake in PfCRT microsomes

^3^H-CQ uptake was measured as described previously [33], with some modifications. Non-specific uptake was determined by pre-incubation with 200-fold unlabelled CQ. Incubation was performed with ^3^H-CQ at 308 nM for 5 min, after which 200-fold unlabelled CQ was added to stop the reaction. Microsomes were then precipitated by the addition of polyethylene glycol (PEG) 8000 and washed twice with accumulation buffer containing 200-fold unlabelled CQ to remove excess ^3^H-CQ. Microsomes were then resuspended in scintillation buffer and agitated overnight. Radioactivity was measured using the LS 5600 Scintillation Counter (Beckman). All data presented are specific uptake.

### Genotyping of strains and isolates

To assess *pfmdr1* polymorphisms, parasite DNA from *in vitro* cultures was extracted with the QIAamp DNA Mini kit (Qiagen) as per the manufacturer's instructions. For *pfcrt* polymorphisms, total RNA was extracted with the RNeasy Mini Kit (Qiagen) and reverse transcription performed with SuperScript III (Invitrogen) as per manufacturers' instructions. Polymerase chain reaction (PCR) mixtures were made with 200 µM of each dNTP, 0.5 µM forward primer, 0.5 µM reverse primer, 0.02 U/µl Phusion DNA polymerase (Thermo Scientific), 6 µl of 5× Phusion HF buffer, and 1 µl of genomic DNA or cDNA to a total reaction volume of 30 µl. Thermocycler parameters were as follows: 98°C for 30 s, followed by 35 cycles of 98°C for 10 s, 60°C for 30 s, and 72°C for 1 min. Primers used for *pfmdr1* sequencing were 5′- ATGGGTAAAGAGCAGAAAGA and 5′- TCCACAATAACTTGCAACAGT, or 5′- GTCAAGCGGAGTTTTTGC and 5′- TATTCTCTGTTTTTGTCCAC. *Pfcrt*-specific primers were 5′- GACGAGCGTTATAGAGAAT and 5′- CTTCGGAATCTTCATTTTCT. PCR products were purified with the QIAquick PCR purification kit as per manufacturer's instructions. Purified PCR products were sequenced by a commercial vendor (AIT Biotech, Singapore). Copy number of *pfmdr1* was assessed by real-time PCR as previously reported [34]. Briefly, reaction mixtures were prepared with TaqMan universal PCR master mix (Applied Biosystems), 5.5 mM MgCl_2_, 300 nM dNTPs, 300 nM each of forward and reverse primers, and 100 nM of the probe. Thermocycler parameters were 95°C for 10 min, then 40 cycles of 95°C for 15 s and 60°C for 1 min. Forward and reverse primers used were 5′- TGCATCTATAAAACGATCAGACAAA and 5′- TCGTGTGTTCCATGTGACTGT respectively, and TaqMan probe was 5′- 6FAM-TTTAATAACCCTGATCGAAATGGAACCTTTG-TAMRA. A reference gene, β-tubulin, was also included; primers were 5′- TGATGTGCGCAAGTGATCC and 5′- TCCTTTGTGGACATTCTTCCTC, while the probe was 5′- VIC-TAGCACATGCCGTTAAATATCTTCCATGTCT-TAMRA. The threshold cycle (C_t_) was analysed by the comparative C_t_ method, based on DNA amplification efficiencies of the *pfmdr1* and β-tubulin genes. *Pfmdr1* copy number was calculated according to the following formula: ΔC_t_  =  C_t_R - C_t_G, where C_t_R is the reference β-tubulin C_t_, and C_t_G is that of *pfmdr1*. Each TaqMan run included three reference DNA samples from clones 3D7, K1, and Dd2 having *pfmdr1* copy numbers of 1, 1, and 3 respectively.

### Statistical analyses

All statistical analyses were performed with SPSS 21. Chemoreversal assays were assessed with Student's t test, 2-tailed. Multiple linear regression was performed with the stepwise method, using the log of IC_50_s and with dummy coded values for the respective amino acid residues.

### Ethics statement

The blood collection protocol for *in vitro* malaria culture was approved by the Institutional Review Board (NUS-IRB Reference Code: 11–383, Approval Number: NUS-1475) of the National University of Singapore (NUS). All participants provided written informed consent. The clinical isolates used were obtained under ethical guidelines in the approved protocol: OXTREC Reference Number 29–09 (Center for Clinical Vaccinology and Tropical Medicine, University of Oxford, Oxford, United Kingdom). Use of field isolates in NUS was in accordance with NUS IRB (Reference Code: 12–369E).

## Results and Discussion

### Validation of CQ_GREEN_ localization and antimalarial activity

**Figure 1:**
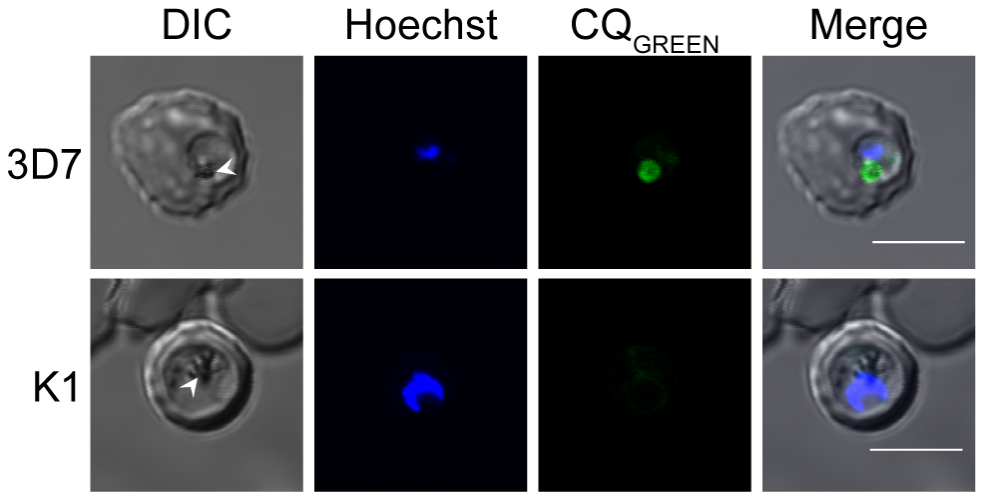
CQ_GREEN_ localization in *P. falciparum* 3D7. Parasites were stained with CQ_GREEN_ and Hoechst and visualized via confocal microscopy under a 100× objective. CQ_GREEN_ accumulates in the DV but also slightly stains parasite cytosol; erythrocyte cytosol is not stained. Arrowheads denote the DV. Scale bars represent 5 µm.

**Figure 2:**
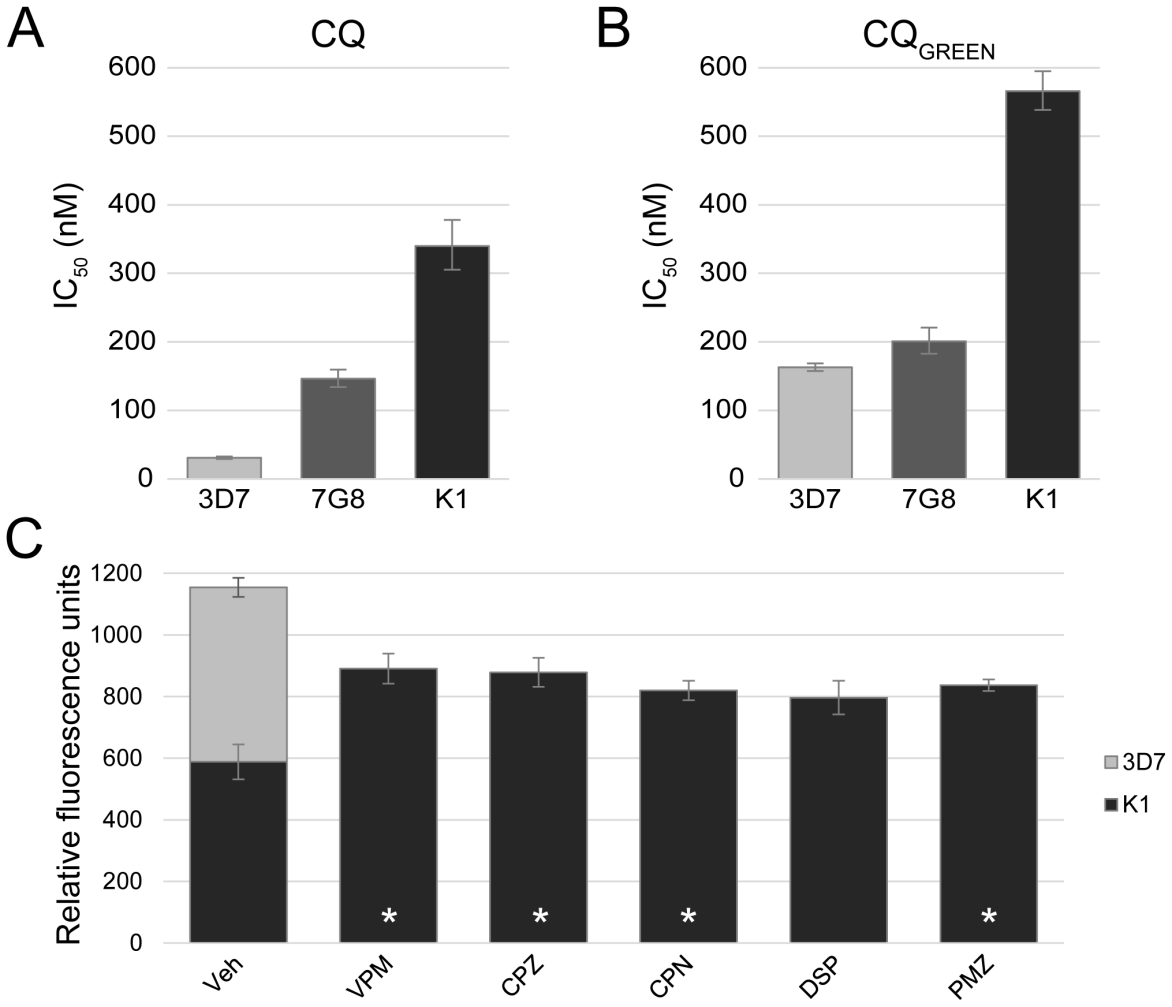
Validation of CQ_GREEN_ activity and uptake in laboratory strains. (A, B) CQ_GREEN_ IC_50_s recapitulates CQ IC_50_s in three *P. falciparum* laboratory strains, 3D7, 7G8 and K1. 3D7 is a CQ-susceptible strain, 7G8 is moderately resistant, while K1 is highly CQ-resistant. Data shown are geometric means from at least 3 experiments. As the error from IC_50_s are not symmetrical, error bars indicate 95% confidence intervals instead of the standard error of the mean (S.E.M.). (C) CQ_GREEN_ uptake as measured by fluorescence is increased in CQ-resistant K1 when pretreated with chemoreversal agents. 3D7 exhibits highest uptake of CQ_GREEN_. Data are means from at least 3 experiments; error bars are S.E.M. Veh: vehicle control; VPM: verapamil; CPZ: chlorpromazine; CPN: chlorpheniramine; DSP: desipramine; PMZ: promethazine. *: p<0.05.

CQ is generally believed to accumulate in the DV as a result of ion-trapping [35], [36]. Confocal microscopy was therefore performed to ascertain the localization of CQ_GREEN_ in the parasite. Cultures of *P. falciparum* 3D7 were co-stained with Hoechst dye and CQ_GREEN_, revealing a preferential accumulation of CQ_GREEN_ in the parasite DV (Fig. 1). Interestingly, CQ_GREEN_ fluorescence was also observed in the parasite cytosol but not in the erythrocyte cytosol. As CQ_GREEN_ is a CQ analog, the antimalarial potency of CQ_GREEN_ should be similar to that of CQ. To assess this, reinvasion IC_50_s of CQ and CQ_GREEN_ on the laboratory strains 3D7, 7G8 and K1 were determined. CQ_GREEN_ showed the same general trend of antimalarial activity as CQ, in that it is most potent against 3D7, followed by 7G8, then K1 (Fig. 2A, 2B).

### CQ_GREEN_ fluorescence as a proxy for CQ uptake in parasites

Next, we determined if CQ_GREEN_ uptake by the highly CQ-resistant strain K1 can be increased by pre-treatment with chemosensitizers. Verapamil, chlorpromazine, chlorpheniramine, desipramine, and promethazine have previously been reported to reverse CQ resistance and increase CQ uptake in CQ-resistant strains [37]–[41]. CQ-sensitive 3D7 was included as a reference for complete reversal. All reversal agents except desipramine induced a significant increase in CQ_GREEN_ fluorescence (Fig. 2C). Desipramine is in fact a less potent reversal agent compared to verapamil when tested in the resistant strain Dd2, and two CQ-resistant field isolates [42]; this may explain why desipramine's effect on CQ_GREEN_ uptake did not achieve statistical significance. Taken together with the CQ_GREEN_ IC_50_ data, we believe that the reversibility of CQ_GREEN_ uptake by known chemoreversal agents suggests that CQ_GREEN_ shares similar structural properties with CQ.

### Uptake of CQ_GREEN_ in microsomes bearing PfCRT

**Figure 3:**
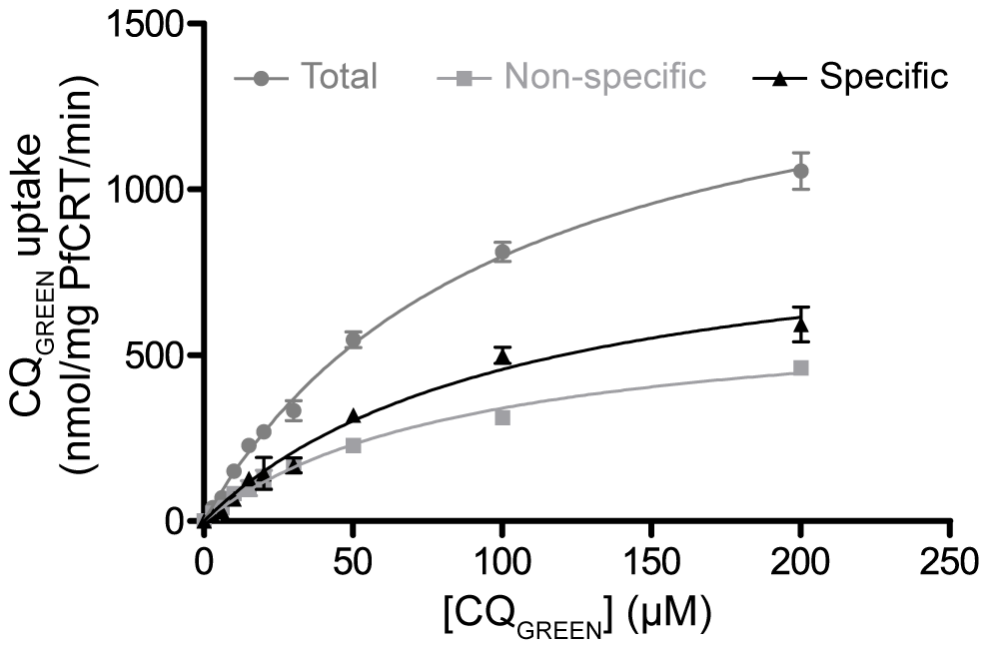
CQ_GREEN_ uptake by Dd2 PfCRT microsomes. Total uptake of CQ_GREEN_ was measured at various CQ_GREEN_ concentrations in microsomes carrying Dd2 PfCRT. Non-specific uptake of CQ_GREEN_ was measured with pre-treatment of excess unlabelled CQ. Specific uptake was estimated as the difference between total and non-specific uptake. V_max_ and K_m_ of the specific uptake was 938.5 nmol/mg PfCRT/min and 105.1 µM respectively. Data are means ± S.E.M.; n≥3.

**Figure 4:**
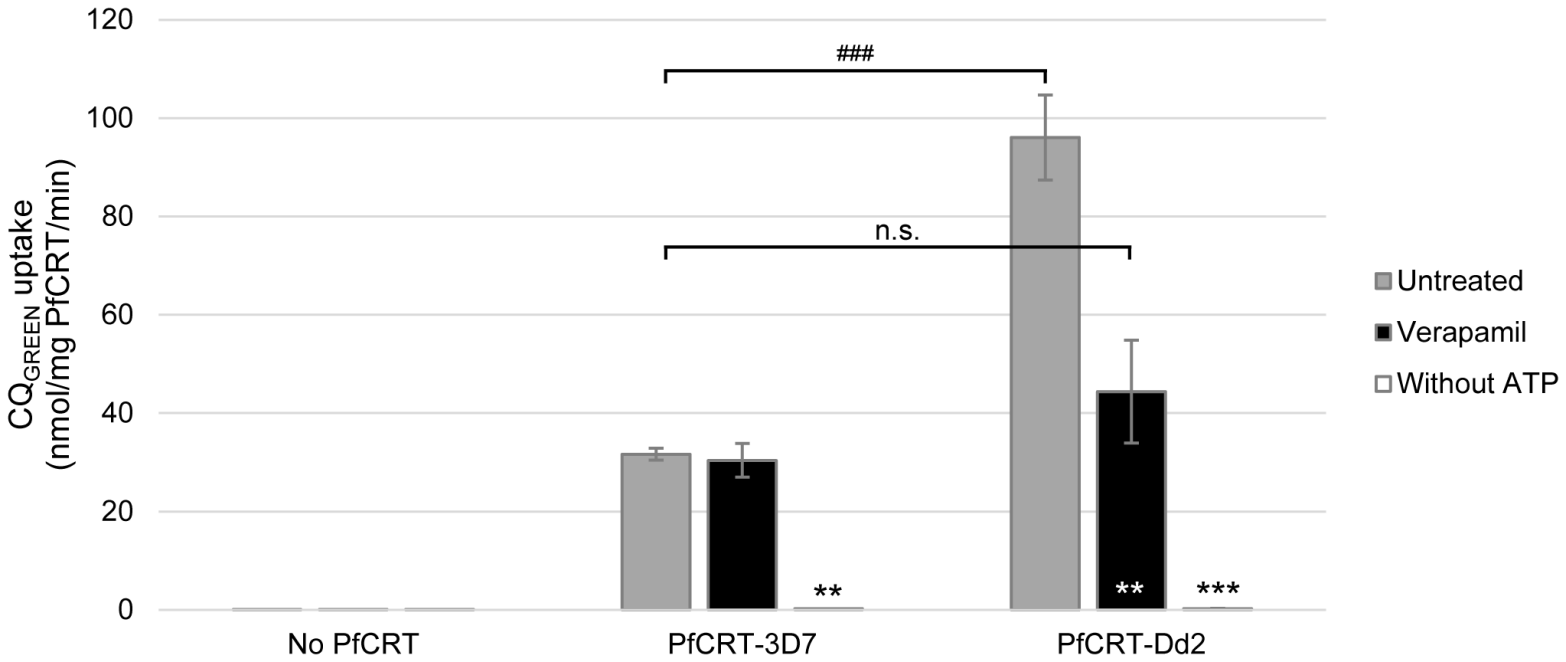
ATP-dependent, verapamil-sensitive uptake of CQ_GREEN_ in microsomes. Yeast microsomes expressing CQ-sensitive or -resistant PfCRT (“PfCRT-3D7” and “PfCRT-Dd2” respectively), or microsomes from plasmid vector control (“No PfCRT”), were incubated with CQ_GREEN_ under different conditions. Preincubation with 150 µM verapamil abrogated CQ_GREEN_ uptake from PfCRT-Dd2 but did not affect uptake in PfCRT-3D7 microsomes. Removal of ATP from buffer abolished CQ_GREEN_ uptake entirely. **, ***: p<0.005 and p<0.001 respectively, against untreated control. ###: p<0.001. N.s.: not significant. Data presented are means ± S.E.M.; n≥3.

**Figure 5:**
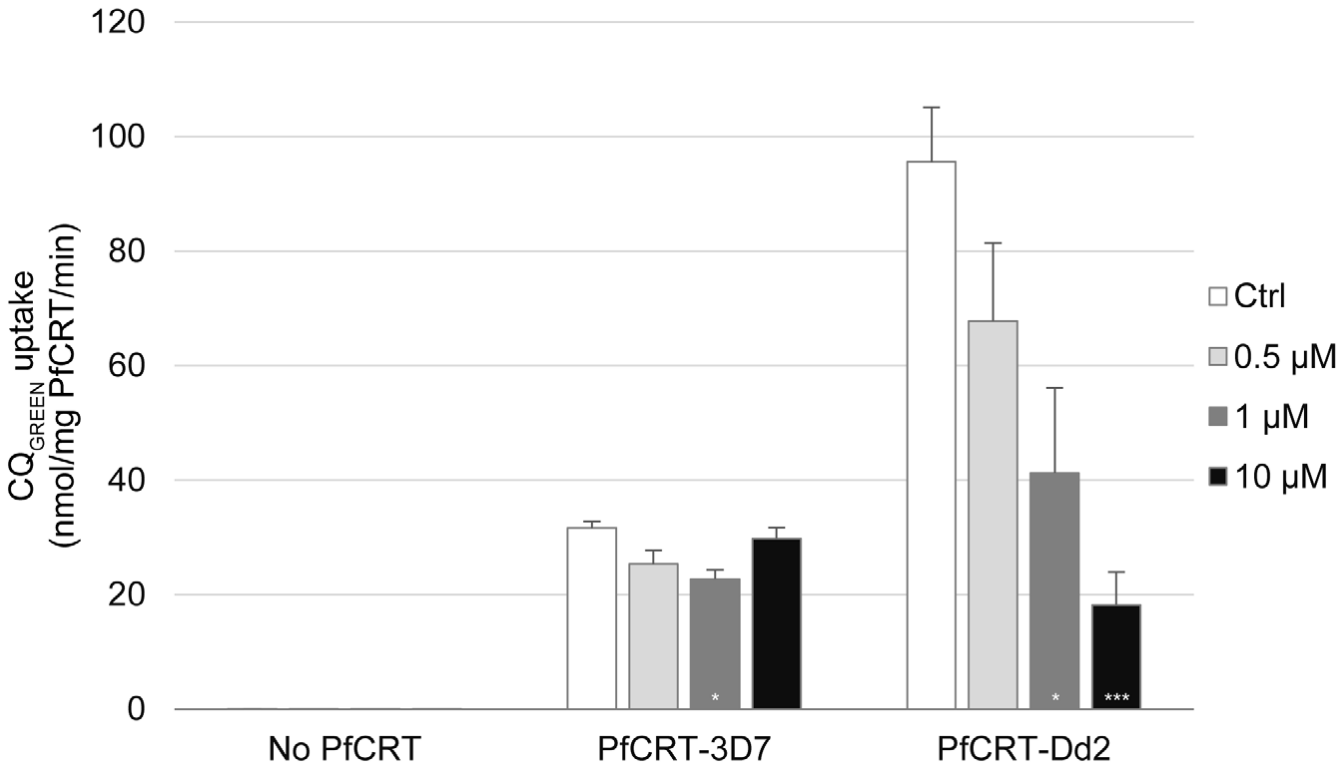
CQ_GREEN_ uptake by resistant-type PfCRT is inhibited by mibefradil in a dose-dependent manner. Microsomes were preincubated with varying concentrations of the PfCRT inhibitor mibefradil prior to addition of CQ_GREEN_. At the highest concentration of 10 µM, mibefradil drastically suppressed CQ_GREEN_ uptake in PfCRT-Dd2 microsomes but had no significant effect on uptake in PfCRT-3D7 microsomes. *, ***: p<0.05 and p<0.001 respectively, against no mibefradil control (Ctrl). Data presented are means ± S.E.M.; n≥3.

**Figure 6:**
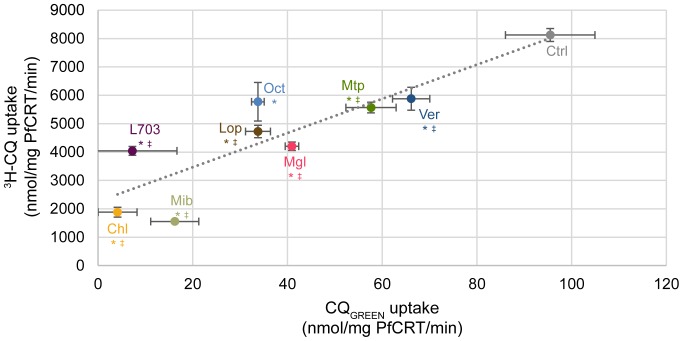
Accumulation of CQ_GREEN_ and ^3^H-CQ in PfCRT-Dd2 microsomes. Microsomes were incubated with 10 µM of chemosensitizers before addition of CQ_GREEN_ or ^3^H-CQ. Ctrl: negative control; Ver: verapamil; Mtp: methiothepin; Mgl: metergoline; Lop: loperamide; Oct: octoclothepin; Mib: mibefradil; L703: L703,606; Chl: chlorprothixene. *: p<0.05, comparing CQ_GREEN_ uptake against control. ^‡^: p<0.05, comparing ^3^H-CQ uptake against control. Data presented are means ± S.E.M.; n≥3.

To test if CQ_GREEN_ can be transported by PfCRT, we have expressed PfCRT in *Pichia pastoris* and used the microsomes derived to study CQ_GREEN_ uptake. Figure 3 shows that CQ_GREEN_ uptake in Dd2 PfCRT-expressing microsomes is specific. At the highest concentration of CQ_GREEN_ used (200 µM), uptake was close to saturated. Michaelis-Menten approximation of CQ_GREEN_ uptake kinetics in Dd2 microsomes (Figure 3) yields a V_max_ and K_m_ of 938.5 nmol/mg PfCRT/min and 105.1 µM respectively, which are approximately 2000 and 500 times higher compared to when ^3^H-CQ was used [33]. Conjugation of CQ with the BODIPY fluorophore may have altered the affinity of PfCRT for the molecule. However, both the high (micromolar) K_m_ and non-saturation of CQ_GREEN_ transport are consistent with a previous report in a *Xenopus* oocyte system using ^3^H-CQ [43]. We have also compared CQ_GREEN_ uptake in microsomes with PfCRT originating from either CQ-sensitive 3D7 or CQ-resistant Dd2. CQ_GREEN_ uptake in Dd2 PfCRT-expressing microsome was 96.07 nmol/mg PfCRT/min, which was about three times that of 3D7 PfCRT (31.64 nmol/mg PfCRT/min) (Figure 4). PfCRT-mediated transport of CQ is thought to be ATP-dependent [25], [44]. Here we demonstrated that removal of ATP completely abolished CQ_GREEN_ uptake in both Dd2 and 3D7 PfCRT microsomes (Figure 4). Verapamil, a known CQ resistance chemosensitizer which has no effect on CQ-sensitive strains, can reverse CQ_GREEN_ uptake in Dd2 PfCRT to almost that of 3D7 level but has almost no effect on 3D7 PfCRT (Figure 4). These results suggest that CQ_GREEN_ is similar to CQ in that (1) it is differentially recognized by CQ-resistant versus CQ-sensitive PfCRT, (2) its uptake by PfCRT is ATP-dependent, and (3) its uptake by CQ-resistant PfCRT is verapamil-reversible. Others have shown that extensive CQ side-chain modifications can render the CQ analogues not transportable by PfCRT and abolish their verapamil sensitivity [45], [46]. Our findings show that the additional BODIPY moiety in CQ_GREEN_ still allows CQ_GREEN_ to be differentially recognized by resistant versus sensitive PfCRT and these transport activities are still sensitive to verapamil. To demonstrate the usefulness of CQ_GREEN_ in screening for PfCRT inhibitors, we found that CQ_GREEN_ uptake by Dd2 PfCRT microsomes can be inhibited by mibefradil, a novel potent chemosensitizer [21], in a dose-dependent manner (Figure 5). Inhibition of CQ_GREEN_ uptake by a panel of known chemoreversal agents was compared to that using ^3^H-CQ (Figure 6). Mean uptake of CQ_GREEN_ was positively correlated with that of ^3^H-CQ (β of 60.24, p = 0.002), showing moderately good agreement between CQ_GREEN_ and ^3^H-CQ uptake (*R* ^2^ = 0.766). However, ^3^H-CQ uptake was roughly 100 times greater than that of CQ_GREEN_.

### Polymorphisms and copy number variation in *pfcrt* and *pfmdr1*

Eight laboratory strains and twelve clinical isolates were sequenced for polymorphisms in PfCRT and Pgh1, the proteins encoded by the genes *pfcrt* and *pfmdr1* respectively. Residues examined were 72, 74, 75, 76, 220, 271, 326, 356, and 371 for PfCRT, and 86, 184, 1034, 1042, 1226, and 1246 for Pgh1. These residues were chosen for analysis as they were previously implicated in modulating multidrug resistance as well as resistance against CQ [47], [48]. Copy number of *pfmdr1* was also determined for each strain and isolate. All clinical isolates showed Dd2-type PfCRT mutations, whereas Pgh1 mutations and *pfmdr1* copy numbers were more varied (Table 1).

**Table 1:** CQ IC_50_s, PfCRT and Pgh1 polymorphisms, and *pfmdr1* copy number.

		PfCRT residue no.									Pgh1 residue no.						*pfmdr1* copy number
Laboratory strains	CQ IC_50_ (nM)	72	74	75	76	220	271	326	356	371	86	184	1034	1042	1226	1246	
**T9/96**	24	C	M	N	K	A	Q	N	I	R	N	Y	S	N	F	D	1
**3D7**	31	C	M	N	K	A	Q	N	I	R	N	Y	S	N	F	D	1
**HB3**	42	C	M	N	K	A	Q	N	I	R	N	**F**	S	**D**	F	D	1
**CS2**	115	C	**I**	**E**	**T**	**S**	**E**	**S**	I	**I**	**Y**	Y	S	N	F	D	3
**T9-94**	146	C	**I**	**E**	**T**	**S**	**E**	**S**	I	**I**	**Y**	Y	S	N	F	D	3
**7G8**	146	**S**	M	N	**T**	**S**	Q	**D**	**L**	R	N	**F**	**C**	**D**	F	**Y**	1
**Dd2**	276	C	**I**	**E**	**T**	**S**	**E**	**S**	**T**	**I**	**Y**	Y	S	N	F	D	3
**K1**	340	C	**I**	**E**	**T**	**S**	**E**	**S**	I	**I**	**Y**	Y	S	N	F	D	1

Clinical isolates	CQ IC_50_ (nM)	72	74	75	76	220	271	326	356	371	86	184	1034	1042	1226	1246	*pfmdr1* copy number
**SMRU0233**	142	C	**I**	**E**	**T**	**S**	**E**	**S**	**T**	**I**	N	Y	S	N	**Y**	D	2
**SMRU0116**	147	C	**I**	**E**	**T**	**S**	**E**	**S**	**T**	**I**	N	Y	S	N	**Y**	D	2
**SMRU0101**	156	C	**I**	**E**	**T**	**S**	**E**	**S**	**T**	**I**	N	Y	S	N	F	D	2
**SMRU1116**	172	C	**I**	**E**	**T**	**S**	**E**	**S**	**T**	**I**	N	Y	S	N	F	D	1
**SMRU0270**	178	C	**I**	**E**	**T**	**S**	**E**	**S**	**T**	**I**	N	Y	S	N	F	D	2
**SMRU0402**	196	C	**I**	**E**	**T**	**S**	**E**	**S**	**T**	**I**	N	Y	S	N	**Y**	D	2
**SMRU1093**	231	C	**I**	**E**	**T**	**S**	**E**	**S**	**T**	**I**	N	Y	S	N	**Y**	D	2
**SMRU0201**	249	C	**I**	**E**	**T**	**S**	**E**	**S**	**T**	**I**	N	Y	S	N	F	D	1
**SMRU0501**	287	C	**I**	**E**	**T**	**S**	**E**	**S**	**T**	**I**	N	**F**	S	N	**Y**	D	1
**SMRU0272**	332	C	**I**	**E**	**T**	**S**	**E**	**S**	**T**	**I**	N	Y	S	N	**Y**	D	3
**SMRU0002**	377	C	**I**	**E**	**T**	**S**	**E**	**S**	**T**	**I**	N	Y	S	N	**Y**	D	1
**SMRU0279**	473	C	**I**	**E**	**T**	**S**	**E**	**S**	**T**	**I**	N	Y	S	N	**Y**	D	1

Bolded residues indicate deviation from 3D7 haplotype. CQ IC_50_s are geometric means of at least 3 measurements.

### Genetic correlates of CQ_GREEN_ uptake

**Figure 7:**
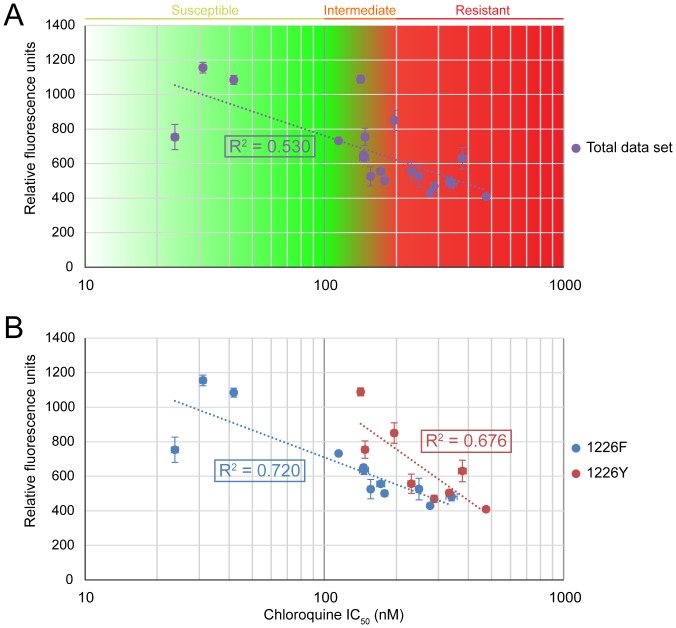
CQ_GREEN_ uptake correlates with CQ resistance. (A) CQ_GREEN_ fluorescence is inversely correlated with CQ logIC_50_. Total data set shows moderate *R* ^2^ of 0.53. (B) When split into subpopulations on the basis of Pgh1 residue 1226, *R* ^2^ is improved. Data shown are means of at least 3 experiments. Error bars represent S.E.M.

All strains and isolates were assayed for CQ_GREEN_ uptake in the trophozoite stage, and their CQ IC_50_s determined with the standard reinvasion assay. For the entire data set, CQ_GREEN_ uptake was inversely correlated to the log of CQ IC_50_, with an *R* ^2^ of 0.53 (Fig. 7A). However, multiple linear regression with sequencing and copy number data revealed that CQ_GREEN_ uptake was significantly correlated with not only CQ logIC_50_ but also a F1226Y substitution in Pgh1 (β of -587.32 and 178.70, p<0.001 and p = 0.024 respectively; adjusted *R* ^2^ of 0.615). None of the other mutations was significantly correlated with CQ_GREEN_ uptake. Separating the data set to two subpopulations improved the *R* ^2^, to 0.72 in the Pgh1 1226F group and 0.676 in the Pgh1 1226Y group (Fig. 7B). It is tempting to conclude that the Pgh1 F1226Y substitution plays a significant causal role in modulating CQ_GREEN_ uptake, given that it is also correlated with resistance to artemisinin, mefloquine and lumefantrine [48] and Pgh1's putative sequestration of cytosol-active drugs in the DV [49]. However, it must be kept in mind that the F1226Y mutation was only detected in the clinical isolates, and given the localized collection of these isolates within a small geographical region, F1226Y is likely to be strongly correlated with other undiscovered mutations. In fact, the PfCRT and Pgh1 haplotypes of the F1226Y mutants examined were identical, apart from SMRU0501 which had an additional Pgh1 Y184F substitution (Table 1). It is notable that for the F1226Y mutants, CQ_GREEN_ uptake could range as high as that of the CQ-susceptible strains (Fig. 7B) while still maintaining CQ resistance. One possible gene modulating CQ_GREEN_ uptake could be *pfmrp*, which has been proposed to efflux drugs across the parasite plasma membrane into the parasitophorous vacuolar lumen [49], which would still contribute to CQ_GREEN_ fluorescence but sequester the drug from its site of action. Knock-out mutants of this gene exhibit increased susceptibility to CQ, quinine, artemisinin, piperaquine, and primaquine [50]. Alternative mechanisms besides efflux of CQ could perhaps also contribute to CQ resistance, such as decreased susceptibility to CQ-induced apoptosis-like cell death [51].

## Conclusions

CQ_GREEN_ is a commercially available fluorescent CQ analog that interacts with the parasite in a similar fashion to CQ. CQ_GREEN_ presents some advantages over traditional radiolabelled CQ in uptake studies: it is safer to handle as it is not radioactive, and its fluorescence properties allows it to be monitored by common fluorescence equipment. Using a defined microsomal platform, we showed that CQ_GREEN_ interacts with PfCRT in a manner similar to CQ. Its use as a predictor of CQ susceptibility is enhanced if residue 1226 of Pgh1 is known. Unlike a typical reinvasion assay which may require 48 h or more, the use of CQ_GREEN_ allows for measurement of CQ susceptibility within several hours. We believe that CQ_GREEN_ could be a valuable tool in future drug discovery projects or used in the identification of factors involved in drug resistance.
